# FGFC1 Selectively Inhibits Erlotinib-Resistant Non-Small Cell Lung Cancer *via* Elevation of ROS Mediated by the EGFR/PI3K/Akt/mTOR Pathway

**DOI:** 10.3389/fphar.2021.764699

**Published:** 2022-01-19

**Authors:** Shike Yan, Bing Zhang, Jingwen Feng, Haigang Wu, Namin Duan, Yamin Zhu, Yueliang Zhao, Shuang Shen, Kai Zhang, Wenhui Wu, Ning Liu

**Affiliations:** ^1^ Department of Marine Bio-Pharmacology, College of Food Science and Technology, Shanghai Ocean University, Shanghai, China; ^2^ School of Life Sciences, Henan University, Kaifeng, China; ^3^ Shanghai Jiao Tong University Affiliated Sixth People’s Hospital, Shanghai, China; ^4^ State Key Laboratory of Oncogenes and Related Genes, Renji-Med X Clinical Stem Cell Research Center, Ren Ji Hospital, School of Medicine, Shanghai Jiao Tong University, Shanghai, China; ^5^ Engineering Research Center of Aquatic-Product Processing & Preservation, Shanghai, China

**Keywords:** FGFC1, non-small cell lung cancer, erlotinib-resistant, mitochondrial dysfunction, ROS, EGFR/PI3K/Akt/mTOR pathway

## Abstract

Non-small cell lung cancer (NSCLC) is one of the most common malignancies in the world. Epidermal growth factor receptor tyrosine kinase inhibitors (EGFR-TKIs) have been used as a first-line treatment for patients harboring with *EGFR* mutations in advanced NSCLC. Nevertheless, the drug resistance after continuous and long-term chemotherapies considerably limits its clinical efficacy. Therefore, it is of great importance to develop new chemotherapeutic agents and treatment strategies to conquer the drug resistance. FGFC1 (Fungi fibrinolytic compound 1), a type of bisindole alkaloid from a metabolite of the rare marine fungi *Starchbotrys longispora.* FG216, has exhibited excellent fibrinolytic and anti-inflammatory activity. However, the potent efficacy of FGFC1 in human cancer therapy requires further study. Herein, we demonstrated that FGFC1 selectively suppressed the growth of NSCLC cells with *EGFR* mutation. Mechanistically, FGFC1 treatment significantly induced the apoptosis of erlotinib-resistant NSCLC cells H1975 in a dose-dependent manner, which was proved to be mediated by mitochondrial dysfunction and elevated accumulation of intracellular reactive oxygen species (ROS). Scavenging ROS not only alleviated FGFC1-induced apoptosis but also relieved the decrease of phospho-Akt. We further confirmed that FGFC1 significantly decreased the phosphorylation of protein EGFR, phosphatidylinositol 3-kinase (PI3K), protein kinase B (Akt), and mammalian target of rapamycin (mTOR) in H1975 cells. Notably, PI3K inhibitor (LY294002) could promote the accumulation of ROS and the expression levels of apoptosis-related proteins induced by FGFC1. Molecular dynamics simulations indicated that FGFC1 can inhibit EGFR and its downstream PI3K/Akt/mTOR pathway through directly binding to EGFR, which displayed a much higher binding affinity to EGFR^T790M/L858R^ than EGFR^WT^. Additionally, FGFC1 treatment also inhibited the migration and invasion of H1975 cells. Finally, FGFC1 effectively inhibited tumor growth in the nude mice xenograft model of NSCLC. Taken together, our results indicate that FGFC1 may be a potential candidate for erlotinib-resistant NSCLC therapy.

## Introduction

Lung cancer has become the leading cause of cancer-related deaths worldwide. Non-small cell lung cancer (NSCLC) as the most common type of cancer accounts for about 80–85% of all lung cancer cases, which makes it a global health concern (Ke et al., 2015; Siegel et al., 2020). Compared with the steady improvement in survival rates for most other types of cancer, advances in treatments for NSCLC have made relatively little progress, and the 5-year survival rates of NSCLC patients remains less than 20% (Cheng et al., 2016).

Somatic mutations of the Epidermal growth factor receptor (*EGFR*) gene are major drivers of NSCLC development and are detected in approximately 30–65% of NSCLC patients in Asia (Paez et al., 2004). The most common mutations of the *EGFR* gene found in NSCLC are deletions in exon 19 and the L858R mutation in exon 21, resulting in activation of EGFR and its downstream signaling pathways, such as phosphatidylinositol 3-kinase (PI3K)/protein kinase B (Akt)/mammalian target of rapamycin (mTOR), which has been proved to play an important role in the development, differentiation, survival and drug resistance of NSCLC (Wee and Wang, 2017). Thus, selective targeted EGFR tyrosine kinase inhibitors (TKIs) are widely developed and used for the treatment of NSCLC (Hanahan and Weinberg, 2011), which has remarkable therapeutic effects against NSCLC patients with *EGFR* mutations. For example, first-generation EGFR-TKIs erlotinib (Minna and Dowell, 2005) and gefitinib (Cohen et al., 2003) showed an encouraging response rate to NSCLC with *EGFR* mutations (exon 19 deletions and L858R point mutations). Unfortunately, more than 80% of patients harboring *EGFR* mutation who initially respond to EGFR-TKI will gradually develop acquired resistance within about 1 year of treatment, mostly due to an additional mutation T790M in exon 20 of *EGFR* (Chan et al., 2006). Currently, numerous TKIs have been designed to directly target T790M mutant NSCLC cells, however, there is still a challenge for them to be used clinically owing to their poor selectivity or potential toxicity (Yang and Tam, 2018). Consequently, novel effective therapeutic agents that can overcome EGFR-TKI resistance should be identified and developed to treat NSCLC, which will ultimately prolong the overall survival time of NSCLC patients.

An essential step for killing resistant NSCLC is mitochondrial dysfunction, which has been proved to play a crucial role in the apoptotic cell death induced by anti-cancer agents (Joseph et al., 2017). Young et al. (2016) reported that Simvastatin can induce mitochondrial apoptosis to overcome gefitinib resistance with *EGFR T790M* mutation NSCLC cells. As well known, reactive oxygen species (ROS) formation is closely related to the mitochondrial pathway, which is confirmed to be the cause and/or consequence of the mitochondrial dysfunction (Kim et al., 2021). High levels of ROS triggers oxidant stress, which leads to cellular damages, tumor progression, and chemotherapy resistance (Brozovic et al., 2010). Several studies have demonstrated that ROS triggers overoxidation of the Met residue of EGFR^T790M^ with direct or indirect involvement in TKI-acquired resistance, and shuts down the EGFR downstream PI3K/Akt survival pathway (Paulsen et al., 2011; Leung et al., 2016). However, the PI3K/Akt signaling pathway can not only be mediated by ROS but also regulate ROS in turn to affect tumor growth. Zhang et al. (2021) have provided clear evidence that dieckol effectively inhibits the human OS MG-63 cells growth by triggering the ROS accumulation via down-regulating the PI3K/Akt signaling pathway (Zhang et al., 2021). In addition, the hyperactivation of PI3K/Akt signaling is usually associated with resistance to different targeted-mediated therapies (Jabbarzadeh Kaboli et al., 2020). Based on the above, inhibiting the PI3K pathway could induce apoptosis and overcome resistance to EGFR-TKI in *EGFR* mutant lung cancer cells (Donev et al., 2011; Gadgeel and Wozniak, 2013; Wang et al., 2019; Lai et al., 2021)*.*

**FIGURE 1:**
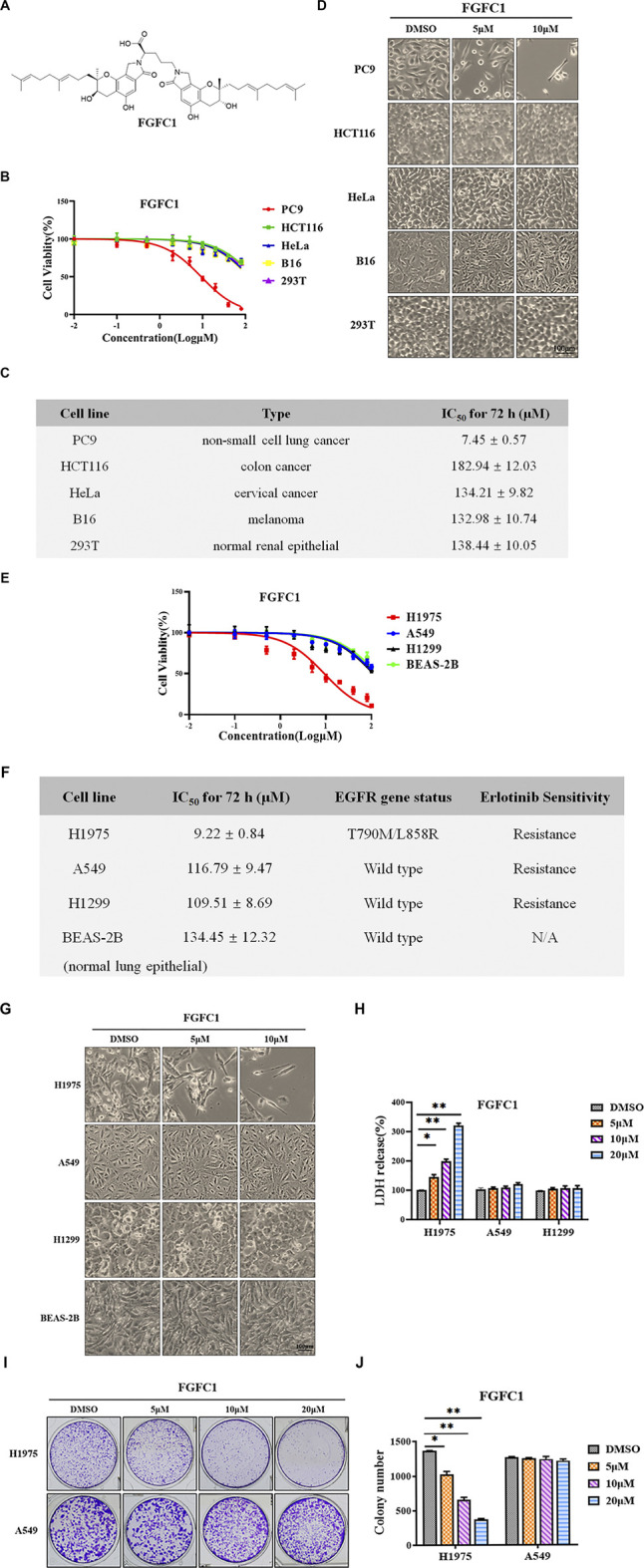
The cytotoxic effects of FGFC1 on different cancer cells. **(A)** Chemical structure of FGFC1 (MW = 869.4947). **(B)** NSCLC cell lines (PC9) were more sensitive to FGFC1 than the other four cell lines for 72 h. PC9, HCT116, HeLa, B16, and 293T cells were incubated with the indicated concentrations of DMSO or FGFC1 (0.01, 0.1, 1, 2, 5, 10, 20, 40, 80, and 100 μM) for 72 h. Cell viability was assessed using CCK-8 assay and shown as relative viability compared to the untreated control. Each test was performed in triplicate. **(C)** The IC_50_ values of FGFC1 in PC9, HCT116, HeLa, B16, and 293T cells were assessed and expressed as the mean ± SD (*n* = 3). **(D)** Above described cells were treated with different concentrations of FGFC1 (0, 5, and, 10 μM) for 72 h. Cell morphology was imaged under microscope (200×). Scale bar = 100 μm. **(E)** FGFC1 selectively inhibited the growth of H1975 erlotinib-resistant NSCLC cells in a dose-dependent manner. H1975, A549, H1299, and BEAS-2B cells were treated with FGFC1 (0.01, 0.1, 1, 2, 5, 10, 20, 40, 80, and 100 μM) for 72 h, respectively. Cell viability was examined by CCK-8 assay and shown as relative viability compared to the untreated control. Each test was performed in triplicate. **(F)** The IC_50_ values of FGFC1, the *EGFR* mutations status, and the erlotinib sensitivity of these four cells were assessed and expressed as the mean ± SD (*n* = 3). **(G)** H1975, A549, H1299, and BEAS-2B cells were treated with different concentrations of FGFC1 (0, 5, and 10 μM) for 72 h. Cell morphology was imaged under microscope (200×). Scale bar = 100 μm. **(H)** FGFC1 remarkably increased the LDH leakage of H1975 cells. The above described cells were incubated with the indicated concentrations of DMSO or FGFC1 (0, 5, 10, and 20 μM) for 48 h. The cytotoxicity of FGFC1 on these cells was examined by the release of LDH. **(I)** FGFC1 significantly inhibited colony formation ability of H1975 cells in a dose-dependent manner. Colony formation of H1975 and A549 cells was monitored after FGFC1 (0, 5, 10, and 20 μM) treatment for 14 days, and photographs of crystal violet stained colonies were depicted. **(J)** The statistical result of **(I)**. All data were represented as the means ± SD for at least three independent experiments. (*p < 0.05 and **p < 0.01 vs the DMSO control).

Up to now, about 63% of the existent anti-tumor agents are natural products (Newman and Cragg, 2016). Recently, marine natural products (MNPs) have been valuable sources for novel drug discovery due to their unique chemical diversity and excellent biological activity (Khalifa et al., 2019). As reported in our previous studies, we obtained a series of MNPs FGFC1, FGFC2, FGFC3, and FGFC4 from a metabolite of the rare marine fungi *Starchbotrys longispora.* FG216 (Wang et al., 2015; Guo et al., 2016). Among them, FGFC1{Fungi fibrinolytic compound (R)-2,5-bis((2R,3R)-2-((E)-4,8-dimethylnona-3,7-dien-1-yl)-3,5-dihydroxy-2-methyl-7-oxo-3,4,7,9-tetrahydropyrano[2,3-e]isoindol-8(2H)-yl)pentanoic acid} (Figure 1A), has been shown to have fibrinolytic activity and anti-inflammatory properties both *in vitro* and *in vivo* (Sawada et al., 2014; Yan et al., 2015; Huang et al., 2018; Suzuki et al., 2018). However, its anti-cancer activity in NSCLC remains unknown. In the present study, we investigated the anti-cancer effects and possible mechanisms of FGFC1 against erlotinib-resistant *EGFR* ^*T790M/L858R*^-driven NSCLC. We found that FGFC1 selectively exhibited significant anti-cancer effects on erlotinib-resistant H1975 NSCLC cells *in vitro* and reduced their tumorigenicity *in vivo*. FGFC1 induced H1975 apoptosis through mitochondrial dysfunction and cumulatively elevated levels of intracellular ROS. These therapeutic efficacies were associated with the binding ability of FGFC1 to EGFR^T790M/L858R^ and inactivation of PI3K/Akt/mTOR signaling pathway, which was important for driving tumorigenesis of NSCLC and TKI resistance. Collectively, our results provide new possibilities that FGFC1 can be used as a lead compound for the treatment of erlotinib-resistant NSCLC with *EGFR* T790M mutation and L858R activating mutation.

## Materials and Methods

### Chemical and Reagents

FGFC1 (≥98% purity) was extracted and purified from the methanol extracts of *Stachybotrys longispora*. FG216, as previously described (Wang et al., 2015; Guo et al., 2016). The medium of FG216 fermented samples was centrifuged and filtered. Marine microorganism precipitation was obtained (200 g), which was added methanol into 2 L. After ultrasonic extraction, centrifuging, and filtering, the sample immersed into methanol was concentrated under vacuum at 40–60°C. The sample was dissolved with 60% saturation using NaCl and adjusted to pH 3.0 with 6N HCl. Then the acid solution was extracted triple with ethyl acetate. The ethyl acetate layer was extracted triple with saturated sodium chloride solution. Finally, the acid solution layer was concentrated in vacuum and dissolved to obtain the crude extract. Then, the extract was subjected to preparative High Performance Liquid Chromatography (HPLC) on an Inertsil PREP-ODS column (22.5 × 250 mm), which was developed at 40°C with a gradient elution of acetonitrile and 0.1% trifluoroacetic acid at a rate of 10 ml/min. After the purified compounds were extracted with ethyl acetate, the fractions that contain the fibrinolytic products were evaporated to remove acetonitrile and trifluoroacetic acid. The chemical structure is shown in Figure 1A. FGFC1 was dissolved in DMSO to make a stock solution, which is convenient for diluting to various concentrations with cell culture medium before using. Erlotinib was purchased from Selleck (Selleck Chemicals, Houston, TX, United States). Dimethyl sulfoxide (DMSO) was purchased from Sigma Aldrich (St. Louis, MO, United States). Dulbecco’s Modified Eagle Medium (DMEM), Roswell Park Memorial Institute (RPMI)-1640 medium, phosphate-buffered saline (PBS) washing buffer, Fetal bovine serum (FBS), Trypsin-EDTA solution, and Penicillin-Streptomycin solution (PS) (100×) were all purchased from Gibco (Carlsbad, CA, United States). Cell Counting Kit-8 (CCK8), Lactate dehydrogenase (LDH) Cytotoxicity Assay Kit, RIPA lysis buffer, crystal violet, ROS assay kit, Mitochondrial membrane potential assay kit with JC-1, GSH and GSSG Assay Kit and LY294002 were purchased from Beyotime (Shanghai, China). *N-Acetyl*-_L_-cysteine (NAC) was purchased from MedChemExpress (Shanghai, China). Annexin V- Fluorescein isothiocyanate (FITC) apoptosis detection kit was obtained from BD Biosciences (San Jose, CA, United States). Bicinchoninic acid (BCA) Protein assay kit was acquired from TIANGEN (Shanghai, China). The cocktail was obtained from Roche (Basel, Lewes, UK). Polyvinylidene fluoride (PVDF) membranes were purchased from Millipore (Billerica, MA, United States). All other chemicals were determined to be of high purity and were purchased from commercial sources.

The following antibodies were used for western blotting. β-actin (Cat#3700) and primary antibodies against cleaved- poly ADP-ribose polymerase (PARP) (Cat#5625), cleaved-caspase3 (Cat#9661), Bcl-2 (D55G8) (Cat#4223), Bax (D2E11) (Cat#5023), cytochrome C (Cat#4272), EGFR (Cat#4267), phospho-EGFR (Y1068) (Cat#2234), PI3K (p110α) (Cat#4249), phospho-PI3K p85 (Tyr458)/p55 (Tyr199) (Cat#4228), Akt (Cat#9272), phospho-Akt (Ser473) (Cat#9271), mTOR (Cat#2983), and phospho-mTOR (Ser2448) (Cat#2971) were all purchased from Cell Signaling Technology (Beverly, MA, United States). Peroxidase-conjugated goat anti-rabbit and mouse secondary antibodies were purchased from Sigma Aldrich (St. Louis, MO, United States).

### Cell Lines and Cell Culture

H1975, PC9, A549, H1299, HeLa, HCT116, B16, BEAS-2B and 293T cells were obtained from the Institute of American Type Culture Collection (ATCC) (Manassas, VA, United States). These cell lines were passaged for less than 6 months after recovery. PC9 cells harbor an exon 19 in-frame deletion (DelE746-A750) of the *EGFR* gene (Lee et al., 2011). H1975 cells harbor two mutations (L858R and T790M) in the *EGFR* gene and are associated with erlotinib resistance (Sudo et al., 2013). H1975, PC9, H1299, HeLa, BEAS-2B and HCT116 cell lines were cultured in RPMI-1640, whereas A549, 293T, and B16 cell lines were cultured in DMEM. All culture media were supplemented with 10% FBS with 100 U/ml penicillin and 100 μg/ml streptomycin, and cells were cultured at 37°C in 5% CO_2_. The cell lines were routinely tested to confirm that they were free of *Mycoplasma*.

### Cell Viability Assays

Cell viability of treated or untreated with FGFC1 was evaluated by CCK-8 assay. Briefly, cells were seeded into 96-well plates at a density of 2–3 × 10^3^ cells per well and allowed to adhere overnight (Zhang et al., 2009; Rong et al., 2019). Cells were treated with different doses of FGFC1 for 72 h, and DMSO served as vehicle control. Each dosage was repeated in triplicate, and three independent experiments were performed. After treatment, 10 μL CCK-8 reagent was added to each well and incubated for an additional 2 h. Finally, the absorbance of each well at 450 nm was measured by a Microplate Reader (BIO-TEK, Inc., Winooski, VT, United States). The IC_50_ value was calculated by GraphPad Prism 8.0 (San Diego, CA, United States) software.

### Lactate Dehydrogenase Release Assay

The cytotoxicity of FGFC1 was analyzed by LDH release assay using LDH Cytotoxicity Assay Kit. NSCLC cells were seeded in a 96-well plate at a density of 1 × 10^4^ cells per well and were treated with different concentrations of FGFC1 (0, 5, 10, and 20 μM). According to the manufacturer’s instructions, after 48 h treatment, the 96-well plate was centrifuged at 400 g for 5 min. Then, the 120 μl of supernatant from each well was transferred to a new 96-well plate and added 60 μl of LDH maximum leakage, following centrifugation. After the 96-well plate was prevented from light for 30 min, the absorbance value was then detected at 490 nm with Microplate Reader (BIO-TEK, Inc., Winooski, VT, United States). The result was evaluated by GraphPad Prism 8.0 (San Diego, CA, United States) software.

### Colony Formation Assay

Colony formation assay was used to examine the long-term effects of FGFC1 on NSCLC cell growth. Cells were seeded in six-well plates at a density of 1,000 cells per well. After attachment overnight, cells were exposed to various concentrations of FGFC1 (0, 5, 10, and 20 μM) with medium changed every 3 days until visible colonies formed. The colonies were washed with cold PBS, then fixed with 4% paraformaldehyde (PFA) and stained by crystal violet for 15 min at room temperature and photographed by a camera. Macroscopic colonies of each well were counted.

### Apoptosis Assays

Cell apoptosis was detected by flow cytometry using an Annexin V-FITC apoptosis detection kit. According to the manufacturer’s instructions, NSCLC cells were seeded in 6-well plates at a density of 1 × 10^5^ cells per well and were treated with FGFC1 (0, 5, 10, and 20 μM) for 48 h, DMSO served as vehicle control. Subsequently, cells were collected and then stained with Annexin V and Propidium Iodide (PI) in 1 × binding buffer at room temperature for 15 min. Finally, stained cells were analyzed using FACSCelesta flow cytometer (BD Biosciences, San Jose, CA, United States). FlowJo software was used to analyze the percentage of cells that undergo apoptosis.

### Mitochondrial Membrane Potential (MMP) Detection

The mitochondrial membrane potential was evaluated using MMP assay Kit with JC-1. H1975, A549, and H1299 cells were seeded into 6-well plates at a density of 1 × 10^5^ cells per well and treated with FGFC1 (0, 10, and 20 μM) for 24 h, DMSO served as vehicle control. The cells were then washed once with PBS and incubated with JC-1 staining solution at 37°C, 5% CO_2_ for 20 min. After incubation, the cells were washed twice and resuspended using the JC-1 assay buffer followed by centrifuge. MMP of FGFC1-treated cells was quantified using FACSCelesta flow cytometer. Cells with red JC-1 aggregates and mitochondrial-collapsed apoptotic cells with green JC-1 monomers were detected in the PI and FITC channels, respectively. All analyses were performed with FlowJo software.

### JC-1 Staining

Changes of MMP were investigated using JC-1 Assay Kit. H1975, A549, and H1299 cells were seeded into 6-well plates at a density of 1 × 10^5^ cells per well and treated with FGFC1 (0, 10, and 20 μM) for 24 h, DMSO served as vehicle control. According to the manufacturer’s instructions, the treated cells were incubated with JC-1 staining solution in a dark for 20 min at 37°C and washed cells with dilution buffer. Then the cellular fluorescence of both JC-1 monomers and aggregates was visualized under fluorescent microscopy (Leica DMI8, Germany).

### ROS Measurement

2,7-Dichlorodihydrofluorescein diacetate (DCFH-DA) was used as ROS probe to detect ROS. NSCLC cells were seeded into 6-well plates at a density of 1 × 10^5^ cells per well and treated with FGFC1 (0, 5, 10, and 20 μM), NAC (5 mM) or LY294002 (20 μM) alone or co-treated with NAC (5 mM) or LY294002 (20 μM) and FGFC1 (10 μM) for 24 h, DMSO served as vehicle control. Then the cells were harvested, centrifuged, and incubated with 10 μM DCFH-DA at 37°C for 30 min. Subsequently, cells were washed twice with PBS. Finally, the mean fluorescence intensity was analyzed using FACSCelesta flow cytometer and data then were evaluated with FlowJo software.

### GSH Intracellular Assay

Intracellular glutathione (GSH) content was measured using the GSH and GSSG Assay Kit according to the manufacturer’s instructions. Briefly, after indicated concentrations of FGFC1 (0, 5, 10, and 20 μM) treatment, NSCLC cells were trypsinized and washed with ice-cold PBS. Next, cells were centrifuged at 1000 rpm for 5 min at 4°C, and then were lysed and vortexed thoroughly in a 96-well plate. 150 μl of the total-glutathione detection solution chromogen was added to the plates, and 10 μl of the diluted prepared samples was added to each well and mixed thoroughly. The plates were equilibrated at 25°C for 5 min. Subsequently, 50 μl of reduced form of nicotinamide-adenine dinucleotide phosphate (NADPH) (0.5 mg/ml) was added to each well. GSH standards (0, 0.5, 1, 2, 5, 10, and 15 μM) were also similarly prepared and assayed at the same time. Finally, the colorimetric intensity of the plates was measured at 412 nm with Microplate Reader (BIO-TEK, Inc., Winooski, VT, United States), and the data were assessed by GraphPad Prism 8.0 (San Diego, CA, United States) software.

### Wound-Healing Assay

A wound-healing assay was performed as previously described (Wang et al., 2013). In brief, NSCLC cells were seeded into 6-well plates and treated with medium containing mitomycin (10 μg/ml) for 3 h to inactivate cell proliferation. The cells were scratched with 200 µL pipette tips and then washed three times with PBS. Complete culture media containing FGFC1 (0, 5, and 10 μM) was subsequently added to allow wound healing. After 24 h of incubation, images of the cells were captured under a microscope equipped with a camera (Nikon, Japan). The migrated cells were counted manually.

### Transwell Invasion Assay

The invasion assay was detected with transwell membranes. Briefly, matrigel diluted in the serum-free medium was applied on top of the transwell membrane and incubated in 5% CO_2_ at 37°C for 4 h. The lower chamber was filled with 800 μl of complete medium containing 20% FBS as chemoattractant. The upper coated chamber was laid over the lower chamber. Then the cells treated with FGFC1 (0, 5, and 10 μM) were suspended in the serum-free medium at a density of 1 × 10^5^/ml and then seeded onto upper chamber wells. The transwell system was incubated at 37°C for 24 h. Later, the transwell insert was carefully removed from the plate, and 600 μl of 4% PFA was added to fix cells. Finally, cells were stained with 0.1% crystal violet solution and observed under an inverted microscope (Olympus Corporation, Japan).

### Western Blotting Analysis

After being treated with different doses of FGFC1, erlotinib, NAC, or LY294002 for 24 h, NSCLC cells were collected on ice and washed twice with PBS. The cell pellets were lysed in RIPA buffer with a complete protease inhibitor cocktail for 10 min on ice followed by centrifugation at 4°C. The concentration of proteins was determined by the BCA Protein kit. Equal amounts of total proteins were resuspended in loading buffer, boiled at 100°C for 5 min, and separated by 12% sodium salt-polyacrylamide gel electrophoresis (SDS-PAGE). Proteins were transferred to PVDF membranes. Then the membranes were blocked with 5% non-fat dry milk in tris-buffered saline and tween 20 (TBST) for 1 h and subsequently incubated with specific primary antibodies (1:1,000) overnight at 4°C. Then the membranes were washed with TBST three times, followed by incubation with the secondary antibodies conjugated with horseradish peroxidase (HRP) (1:10,000) for 1 h. Immunoblots were visualized with the Bio-Rad ChemiDoc XRS system. Quantification was directly performed on the blot using the Image Lab software.

### Molecular Docking Analysis

All the molecular docking simulations were following the previous reports (Yang et al., 2014; He et al., 2016) (https://www.ebi.ac.uk/thornton-srv/software/LIGPLOT/). The computational docking of FGFC1 to EGFR was performed on AutoDock 4.2 software (Morris et al., 2008) for binding mode prediction. Schrödinger software was used for the preparation of the ligand and macromolecular. The 3D structures of EGFR^T790M/L858R^ (PDB ID:3W2R) and EGFR^WT^ (PDB ID:3POZ) was obtained from Protein Data Bank. FGFC1 was docked into the binding site of the EGFR^T790M/L858R^ and EGFR^WT^ with the standard precision scoring mode. In the process of molecular docking, the best binding pose for FGFC1 was conserved for further analysis. The molecular docking results were analyzed using AutodockTools and ligplot+ v1.1.

### Animal Studies

BALB/c nude mice (male, 5–6 weeks old, 10–14 g), supplied by the Jiesijie Experimental Animal Co. Ltd (Shanghai, China), were raised in a pathogen-free and temperature-controlled environment. H1975 cells (5 × 10^6^) were subcutaneously implanted into the right flank of nude mice. Tumor volumes were evaluated by calipers and calculated using the standard formula: volume = 0.5 × (width)^2^ × length. Once tumor volume exceeded approximately 100 mm^3^, mice were randomized into three treatment groups (six mice per treatment group): vehicle (5% DMSO in PBS), FGFC1 (10 mg/kg/d, ip), and erlotinib (10 mg/kg/d, ip) as a control for 21 consecutive days. Mice were treated for 21 days and the tumor volume and body weight were measured after every 3 days. All animal experiments were approved by the Animal Ethical Committee (Permit Number: SHOU-DW-2018-054) of Shanghai Ocean University (Shanghai, China). The experiment was performed according to the NIH guidelines for animal care and use. At the endpoint, the mice were anesthetized, and the tumors were harvested for weight and immunohistochemistry analysis after being separated from the surrounding muscles and dermis.

### Histology and Immunohistochemistry

Immunohistochemical staining was performed by Shanghai RecordBio Co., Ltd. (Shanghai, China). Tumor sections were immunostained with specific anti-EGFR and anti-Ki67 antibodies. The images were captured using a Pannoramic MIDI scanner. The immunoreactive score (IRS) system was applied to evaluate the immunoreactivity of each IHC marker (Remmele and Stegner, 1987). Briefly, IRS = staining intensity × percentage of positive cells. Staining intensity was scored as 0 (negative), 1 (weak), 2 (moderate), and 3 (strong). Percentage of positive cells was scored as 0 (0–5%), 1 (6–25%), 2 (26–50%), 3 (51–75%), and 4 (76–100%). Ten visual fields from different areas of each specimen were chosen randomly for the IRS evaluation, and the average IRS was calculated as the final score.

### Statistical Analysis

All experiments were repeated at least three times and the quantified data were expressed as the mean ± SD unless otherwise indicated. Comparisons were made using one-way ANOVA analysis. *p*-values < 0.05 was considered to be statistically significant. Statistical analyses were performed with GraphPad Prism 8.0 software.

## Results

### FGFC1 Exhibited Different Degrees of Sensitivity Among Various Cancer Cell Lines

The main purpose of this study was to investigate the effects and mechanism of FGFC1 on cancer cells. Therefore, normal renal epithelial cell line (293T), PC9 (NSCLC), HCT116 (colon cancer), HeLa (cervical cancer), and B16 (melanoma) cells were treated with FGFC1 to determine its cellular cytotoxicity on these cells. FGFC1 showed varied anti-proliferative activities against the tested cells (Figure 1B). Interestingly, FGFC1 dramatically decreased the viability of PC9 cells in an apparent dose-dependent manner, but only slight inhibitory effect was observed in HCT116, HeLa, B16, and 293T cells, suggesting that FGFC1 exerted a selective inhibitory effect on PC9 cells. Figure 1C summarized the IC_50_ values of FGFC1 in the 5 cell lines. Compared with the normal cell line and other three cancer cell lines, PC9 was the most sensitive to FGFC1 with an IC_50_ value of 7.45 ± 0.57 μM. Whereas, HCT116, HeLa, B16, and 293T were relatively less sensitive with IC_50_ values of 182.94 ± 12.03 μM, 134.21 ± 9.82 μM, 132.98 ± 10.74 μM, and 138.44 ± 10.05 μM, respectively. PC9 cells are known to harbor an exon 19 in-frame deletion (DelE746-A750) of the *EGFR* gene and are sensitive to erlotinib. As shown in Figure 1D, the morphological observation of cells exhibited that the space between PC9 cells was significantly increased and their appearance had evidently changed. Exposure to a low concentration FGFC1, the pseudopodia of PC9 cells extended and the shape was elongated from the epithelial shape to the spindle shape. The cell morphology visibly changed, and the number of viable cells decreased in a dose-dependent manner. However, barely any morphological changes were observed in other cell lines, indicating that FGFC1 selectively induced PC9 cell death. Collectively, this data indicated that FGFC1 might selectively suppress the growth of NSCLC cells with *EGFR* mutations.

### FGFC1 Selectively Inhibited the Growth of Erlotinib-Resistant NSCLC Cells

We further examined the cytotoxic effect of FGFC1 on a normal lung epithelial cell line (BEAS-2B) and three NSCLC cell lines with different *EGFR* status, consisting of two NSCLC cell lines (A549 and H1299) with wild type *EGFR* and another erlotinib-resistant NSCLC cell line H1975 harboring *EGFR* double mutations (L858R/T790M). Interestingly, FGFC1 exhibited substantially stronger cytotoxicity in erlotinib-resistant H1975 cells (IC_50_ = 9.22 ± 0.84 μM) than A549 (IC_50_ = 116.79 ± 9.47 μM), H1299 (IC_50_ = 109.51 ± 8.69 μM), and BEAS-2B (134.45 ± 12.32 μM) (Figures 1E,F). When exposed to FGFC1, as observed in PC9 cells, H1975 cells also showed significant morphological alterations under a phase-contrast microscope. The viable cell numbers decreased in a dose-dependent manner as well. On the other hand, no remarkable changes were seen in *EGFR* wild type cells including A549, H1299 and BEAS-2B cells (Figure 1G). In addition, we also found that FGFC1 treatment remarkably increased the LDH leakage of H1975 cells in a dose-dependent manner (100.67, 145.68, 198.51, and 321.96% following 48 h treatment with FGFC1, respectively), not in A549 and H1299 cells (Figure 1H). Furthermore, we examined the effect of FGFC1 on cell colony formation. The results from experimental colony formation assays indicated that FGFC1 was able to reduce the number and size of the colonies of H1975 cells in a dose-dependent manner compared with A549 cells (Figures 1I,J). Consequently, these results demonstrated that except for erlotinib-sensitive PC9 cells, FGFC1 could selectively inhibit the growth of erlotinib-resistant H1975 NSCLC cells, which harbors L858R/T790M double mutation of *EGFR*. Due to T790M might be the most important mutation, leading to the development of TKI resistance (Rajappa et al., 2019), next we mainly evaluated the effects of FGFC1 on *EGFR* ^*T790M*^ mutant NSCLC cells.

### FGFC1 Selectively Triggered Apoptosis in H1975 Erlotinib-Resistant NSCLC Cells

**FIGURE 2:**
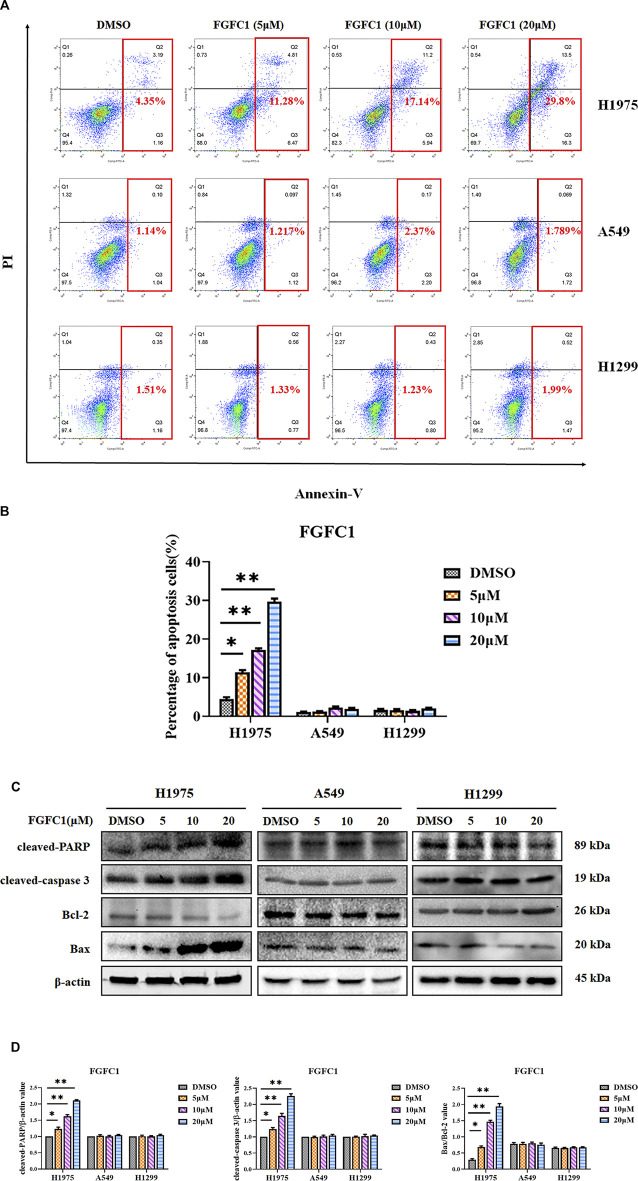
FGFC1 selectively induced apoptosis in erlotinib-resistant NSCLC cells. **(A)** FGFC1 significantly enhanced H1975 cells death. H1975, A549 and H1299 cells were incubated with the indicated concentrations of FGFC1 (0, 5, 10, and 20 μM) for 48 h, and the cell apoptosis rates were detected by the Annexin-V FITC/PI staining assay. **(B)** The statistical result of **(A)**. **(C)** H1975, A549 and H1299 cells were treated with different concentrations of FGFC1 (0, 5, 10, and 20 μM) for 24 h. Cleaved-PARP, cleaved-caspase 3, Bax and Bcl-2 protein levels were examined by Western blotting. β-actin were detected as the endogenous loading control, accordingly. **(D)** The statistical result of **(C)**. All data were represented as the means ± SD for at least three independent experiments (*p < 0.05 and **p < 0.01 vs the DMSO control).

Given the fact that FGFC1 treatment appeared to enhance cell death, especially for the H1975 erlotinib-resistant NSCLC cells when they were treated with high concentrations of FGFC1, we further determined whether this compound caused apoptosis of cells. As presented in Figure 2A, compared to the *EGFR* wild type cells A549 and H1299, the application of FGFC1 had a significant dose-dependent effect upon increasing the apoptotic proportion of H1975 cells. A distinct increase was observed in the percentage of H1975 apoptotic cells (4.35, 11.28, 17.14, and 29.8% following 48 h treatment with FGFC1, respectively) (Figure 2B). In addition, the expression levels of apoptosis-related protein PARP, caspase 3, Bax, and Bcl-2 from these cells were examined by immunoblotting analysis. Our results suggested that FGFC1 dose-dependent promoted the expression levels of cleaved caspase-3, PARP, and Bax, while it markedly dose-dependent inhibited the expression levels of Bcl-2. (Figures 2C,D). In contrast, limited effects were observed in *EGFR* wild type A549 and H1299 cells, which confirmed the selective inhibitory effects of FGFC1 on apoptosis in H1975 cells. These results clearly suggested that FGFC1 effectively induced erlotinib-resistant H1975 cell death by promoting their apoptosis.

### FGFC1 Enhanced Mitochondrial Dysfunction and ROS Generation to Induce Apoptosis in H1975 Erlotinib-Resistant NSCLC Cells

**FIGURE 3:**
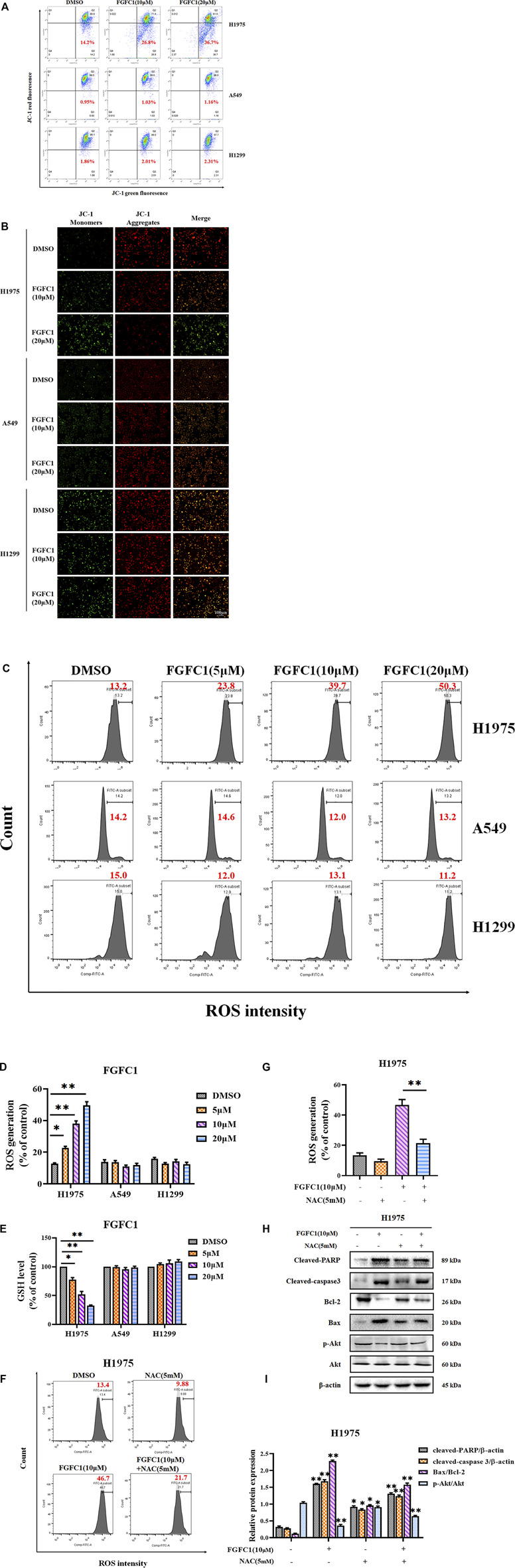
FGFC1 induced apoptosis in erlotinib-resistant NSCLC cells via mitochondrial dysfunction and accumulation of ROS. **(A)** FGFC1 significantly downregulated the MMP level. H1975, A549 and H1299 cells were treated with FGFC1 at different concentrations (0, 10 and 20 μM) for 24 h. The treated cells were stained with JC-1 dye and then analyzed by Flow cytometry. Cell populations indicating JC-1 aggregates (red) were in the upper right quadrant and JC-1 monomers (green) were in the lower right part. **(B)** The treated cells stained with JC-1 were pictured by fluorescence microscope to estimate the alteration in MMP level (scale bar = 100 μm). **(C)** FGFC1 significantly enhanced ROS production in H1975 cells. H1975, A549 and H1299 cells were incubated with the indicated concentrations of FGFC1 (0, 5, 10, and 20 μM) for 24 h, followed by ROS measurement. Representative flow cytometry histograms displaying levels of fluorescent DCFH-DA in cells were presented. **(D)** Statistical analysis of the percentage of ROS generation. **(E)** FGFC1 induced GSH depletion in H1975 cells. The above described cells were treated with various doses of FGFC1 (0, 5, 10, and 20 μM) for 24 h, and then the cellular GSH content was measured using an Assay Kit. **(F)** H1975 cells were pretreated with or without NAC (5 mM) for 2 h, then treated with FGFC1 (10 μM) and NAC (5 mM) alone or in combination for 24 h. Intra cellular ROS were measured by flow cytometry after 10 μM DCFH-DA staining. **(G)** Statistical analysis of the percentage of ROS generation. **(H)** H1975 cells were pretreated with or without NAC (5 mM) for 2 h, then treated with FGFC1 (10 μM) and NAC (5 mM) alone or in combination for 24 h. Cleaved-PARP, cleaved-caspase 3, Bax, Bcl-2 and p-Akt protein expression were evaluated by Western blotting. β-actin was detected as the endogenous loading control, accordingly. **(I)** The statistical result of **(H)**. All data were represented as the means ± SD for at least three independent experiments. (*p < 0.05 and **p < 0.01 *vs* the DMSO control).

Considering the pivotal role of mitochondria in orchestrating the apoptotic pathway, we investigated the effect of FGFC1 on the MMP. The decrease of MMP was a detection index in the early phase of apoptosis and indicated mitochondrial dysfunction. Reduced red fluorescence and increased green fluorescence were regarded as the MMP collapsed. As shown in Figure 3A, compared with *EGFR* wild type A549 and H1299 cells, FGFC1 induced a significant loss of MMP in H1975 cells in a dose-dependent manner. Additionally, fluorescence results confirmed that H1975 cells treated with FGFC1 displayed a clear shift in JC-1 staining of the mitochondria from red to green in a dose-dependent manner (Figure 3B). Moreover, FGFC1 incubation could dose-dependently increase the protein level of cytochrome C in H1975 cells (Supplementary Figure 1). These results signified that FGFC1 could induce mitochondrial dysfunction in H1975 erlotinib-resistant cells. Next, we examined the changes of cellular ROS levels in the above cell lines. As expected, the ROS levels were increased dose dependently after FGFC1 treatment in H1975 cells (13.2, 23.8, 39.7, and 50.3%, respectively) compared with control A549 and H1299 cells. (Figures 3C,D). Additionally, excessive intracellular ROS can deplete cellular GSH (Rahman et al., 2019). Figure 3E showed that FGFC1 indeed reduced the GSH content in H1975 cells dose dependently (100, 77.8, 52.37, and 32.67% following 24 h treatment with FGFC1, respectively), whereas the GSH depletion was not significantly observed in A549 and H1299 cells. Finally, to determine the role of ROS in FGFC1-treated cellular apoptosis, we pre-incubated NSCLC cells with the ROS scavenger NAC to demonstrate the effect of FGFC1 treatment and determine whether NAC could attenuate apoptosis induced by FGFC1. The result of flow cytometry showed that NAC significantly inhibited the accumulation of ROS induced by FGFC1 in H1975 cells (Figures 3F,G). Bcl-2, Bax, cleaved-caspase 3, and PARP protein expression were detected by western blot assay. As shown in Figures 3H,I, suppression effects on Bcl-2 and the apoptotic effects (indicated by the levels of cleaved-caspase 3, PARP, and Bax) were attenuated in the NAC and FGFC1 combined treatment group compared with the FGFC1 treatment alone group. Additionally, our western blot analysis also revealed that FGFC1 inhibited the phosphorylation of Akt, which has been proved to be suppressed by ROS (Palanivel et al., 2014). And this phosphorylation suppression effect was attenuated in the NAC and FGFC1 combined treatment group (Figures 3H,I), which indicated that FGFC1 might trigger anti-proliferation effect via Akt signaling pathway. Consequently, we concluded that FGFC1-treatment-induced cell apoptosis was associated with mitochondrial dysfunction and ROS production in NSCLC cells, and Akt signaling was involved in this process.

### EGFR/PI3K/Akt/mTOR Signaling Pathway Involved in FGFC1-Induced Cell Apoptosis

**FIGURE 4:**
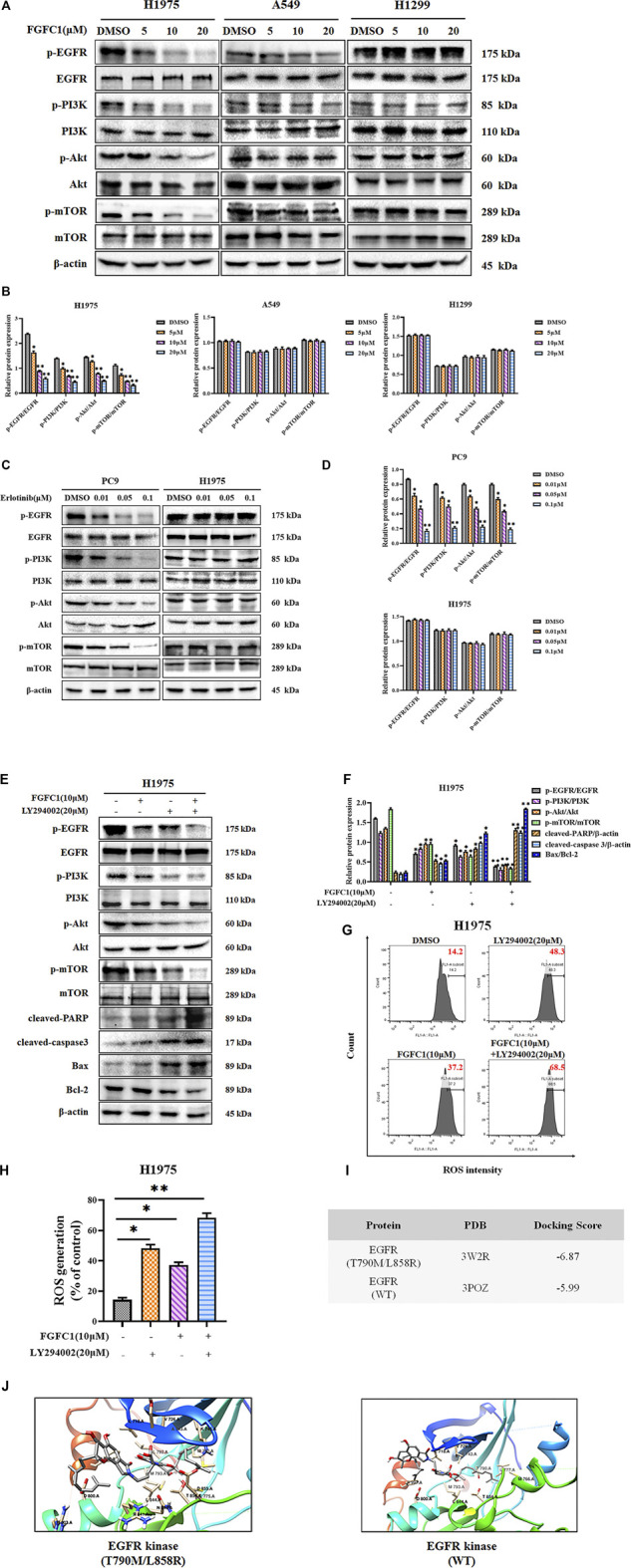
FGFC1 meditated apoptosis through the EGFR pathway against erlotinib-resistant NSCLC cells. **(A)** FGFC1 remarkably suppressed the phosphorylation of EGFR and its downstream targets in H1975 cells. H1975, A549 and H1299 cells were treated with the indicated concentrations of FGFC1 (0, 5, 10, and 20 μM) for 24 h. The expression of p-EGFR, EGFR, p-PI3K, PI3K, p-Akt, Akt, p-mTOR, and mTOR was examined by Western blotting. β-actin was detected as the endogenous loading control. **(B)** The statistical result of **(A)**. **(C)** H1975 and PC9 cells were treated with a range of concentrations of erlotinib (0, 0.01, 0.05, and 0.1 μM) for 24 h, the cell lysates were subjected to Western blotting with the indicated antibodies. **(D)** The statistical result of **(C)**. **(E)** H1975 cells were treated with FGFC1 (10 μM) and LY294002 (20 μM) alone or in combination for 24 h. The expression of p-EGFR, EGFR, p-PI3K, PI3K, p-Akt, Akt, p-mTOR, mTOR, cleaved-caspase3, cleaved-PARP, Bax, and Bcl-2 was evaluated by Western blotting. β-actin was detected as the endogenous loading control, accordingly. **(F)** The statistical result of **(E)**. **(G)** H1975 cells were treated with FGFC1 (10 μM) and LY294002 (20 μM) alone or in combination for 24 h. Intra cellular ROS were measured by flow cytometry after 10 μM DCFH-DA staining. **(H)** Statistical analysis of the percentage of ROS generation. **(I)** Docking score of FGFC1 with EGFR^T790M/L858R^ and EGFR^WT^. **(J)** The binding mode of FGFC1 docked into EGFR^T790M/L858R^ and EGFR^WT^, respectively. Data shown were representative of three independent experiments. All data were represented as the means ± SD for at least three independent experiments. (*p < 0.05 and **p < 0.01 vs the DMSO control).

Due to the selective inhibition of FGFC1 on H1975 erlotinib-resistant NSCLC cells and its inhibition on the phosphorylation of Akt, we tested the activation of EGFR and its downstream signaling pathway PI3K/Akt/mTOR after FGFC1 treatment (Jorissen et al., 2003; Seshacharyulu et al., 2012). Firstly, cells were treated either with FGFC1 or erlotinib for 24 h. As shown in Figures 4A,B, FGFC1 dose-dependently suppressed the phosphorylation of EGFR, PI3K, Akt, and mTOR in H1975 cells, while the levels of total EGFR, PI3K, Akt, and mTOR remained unaltered. Meanwhile, as a parallel control, we also examined the protein levels of these phosphorylation kinases in A549 and H1299 cells, but no evident inhibition was observed. As expected, erlotinib significantly inhibited the phosphorylation of EGFR, PI3K, Akt, and mTOR in PC9 cells, whereas there is no obvious effect in H1975 cells (Figures 4C,D). Furthermore, we used an inhibitor of the PI3K/Akt/mTOR signaling pathway, LY294002, in order to determine whether or not FGFC1 was able to inhibit the PI3K/Akt/mTOR signaling pathway. H1975 cells were exposed to FGFC1 and LY294002 alone or in combination for 24 h. The results clarified that co-treatment with FGFC1 and LY294002 has significantly suppressed Bcl-2 and the phosphorylation of EGFR, PI3K, Akt, and mTOR than with either drug alone group. Conversely, a significant increase in the expression of cleaved-caspase 3, cleaved-PARP, and Bax protein was observed in the combination treatment group (Figures 4E,F). Meanwhile, we also evaluated the levels of ROS in H1975 cells after treatment with FGFC1 and LY294002 alone or in combination for 24 h. As presented in Figures 4G,H, compared with the FGFC1 or LY294002 treatment alone group, the accumulation of ROS was significantly increased in the FGFC1 and LY294002 combined treatment group. Collectively, LY294002 markedly enhanced the inhibitory effect of FGFC1 on the phosphorylation of EGFR, PI3K, Akt, mTOR, and increased FGFC1-induced ROS generation and cell apoptosis. Therefore, we suggested that FGFC1 induced apoptosis and death in H1975 erlotinib-resistant NSCLC cells through the EGFR/PI3K/Akt/mTOR signaling pathway.

### Molecular Docking Predicted the Potential Binding of FGFC1 to EGFR^T790M/L88R^

To identify the direct target of FGFC1, molecular docking was performed to investigate the binding mode of FGFC1 with EGFR^T790M/L858R^ and EGFR^WT^. The molecular docking scores of FGFC1 in various proteins were -6.87 kcal/mol and −5.99 kcal/mol respectively (Figure 4I). As shown in Figure 4J, the hydrophobic groups of FGFC1 bound deeply with the hydrophobic pocket of EGFR^T790M/L858R^, and the interaction complex could be stabilized through binding with Leu718 of EGFR^T790M/L858R^ by a hydrogen bond. Meanwhile, FGFC1 experienced favorable hydrophobic and van der Waals interactions with residues Asp855, Lys745, Val726, Leu788, Leu844, Cys775, Ala743, Met790, Met793 Thr854, and Gln791 in the active pocket, which could further stabilize the protein-ligand complex. The above results suggested that FGFC1 possessed a preferential binding ability to EGFR^T790M/L858R^.

### FGFC1 Prevented H1975 Cell Migration and Invasion

**FIGURE 5:**
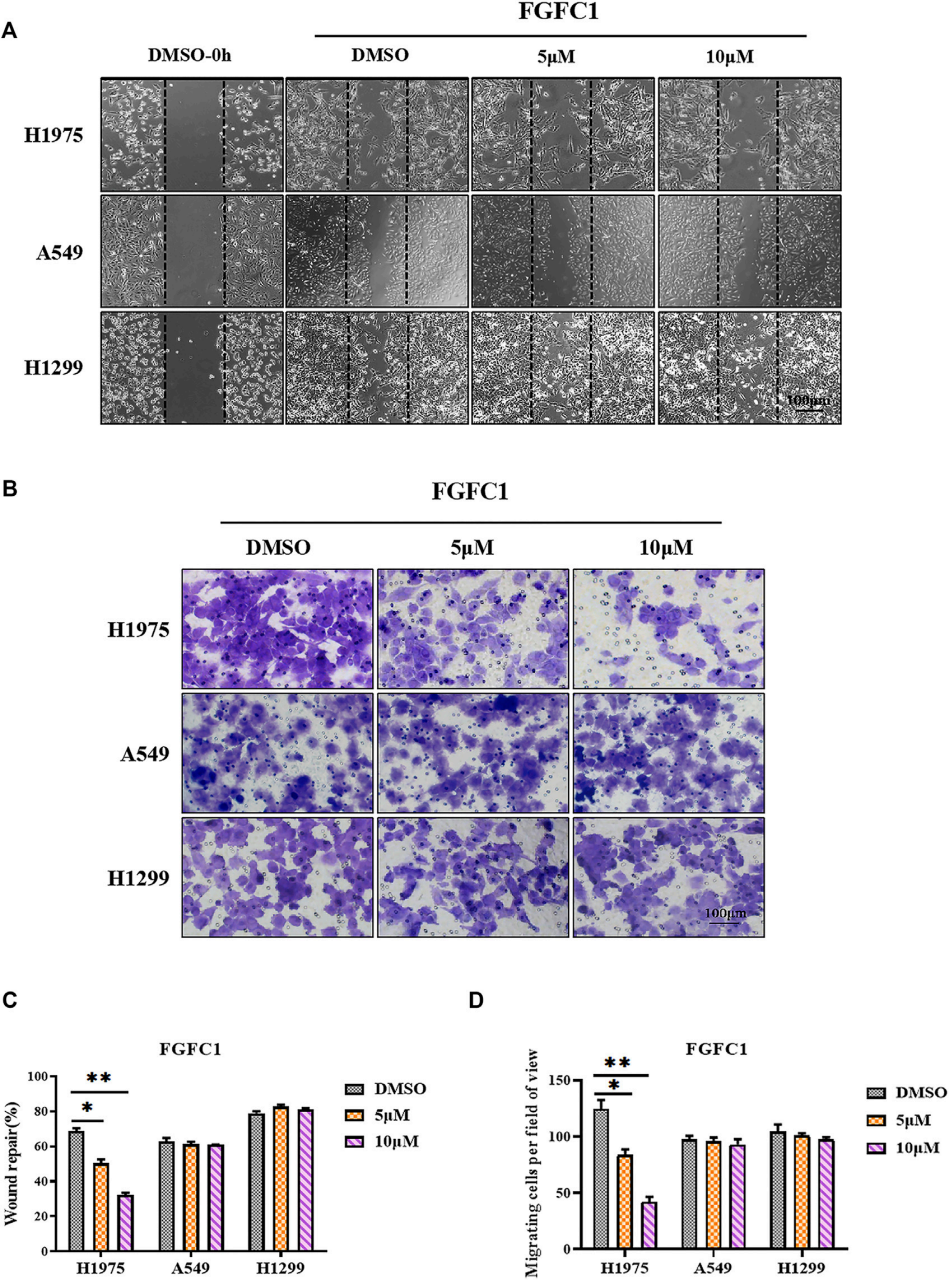
FGFC1 selectively inhibited the migration and invasion of H1975 NSCLC cells. **(A)** The migration of H1975, A549 and H1299 cells was evaluated using the scratch wound-healing assay after cells treated with indicated concentrations of FGFC1. NSCLC cells were seeded in 6-well plates and were treated with the indicated dose of FGFC1 (0, 5, and 10 μM) for 24 h. Images of the results were obtained under microscope (200×) (scale bar = 100 μm). The migrated cells were quantified manually. **(B)** The invasion activity of H1975, A549 and H1299 cells treated with FGFC1 were measured by transwell invasion assay. NSCLC cells were seeded in 24-well plates and were treated with the indicated concentrations of FGFC1 (0, 5, and 10 μM). After 24 h, the invasive cells were stained with crystal violet, and photos were taken under microscope (200×). The invasion activity of these cells was measured by counting the staining positive cells (scale bar = 100 μm). **(C)** Quantification of the data in **(A)**. **(D)** Quantification of the data in **(B)**. All data were represented as the means ± SD for at least three independent experiments. (*p < 0.05 and **p < 0.01 vs the DMSO control).

Tumor cell migration and invasion are vital steps in the process of tumor metastasis, which leads to the death of tumor patients. Thus, we next studied whether FGFC1 was able to affect erlotinib-resistant NSCLC cell migration or cell invasion. Cells were treated with various concentrations of FGFC1 for 24 h. As shown in Figure 5A, the wound-healing assay showed that FGFC1 significantly suppressed the migration capacity of H1975 cells in a dose-dependent manner (Figures 5A,C). While at the same concentration gradient, FGFC1 exhibited a slight inhibition in A549 and H1299. Then, the effect of FGFC1 on NSCLC cells invasion was further examined using a transwell assay, and similar results were observed. The application of FGFC1 had a significant dose-dependent effect upon inhibiting H1975 cells invasion. However, there was still no significant inhibitory effect in A549 and H1299 (Figures 5B,D). Collectively, this data suggested that FGFC1 could suppress the migration and invasion of H1975 cells, which might contribute to the treatment of erlotinib-resistant NSCLC with *EGFR* T790M mutation and L858R activating mutation.

### FGFC1 Suppressed Tumor Growth in H1975 Xenografts

**FIGURE 6:**
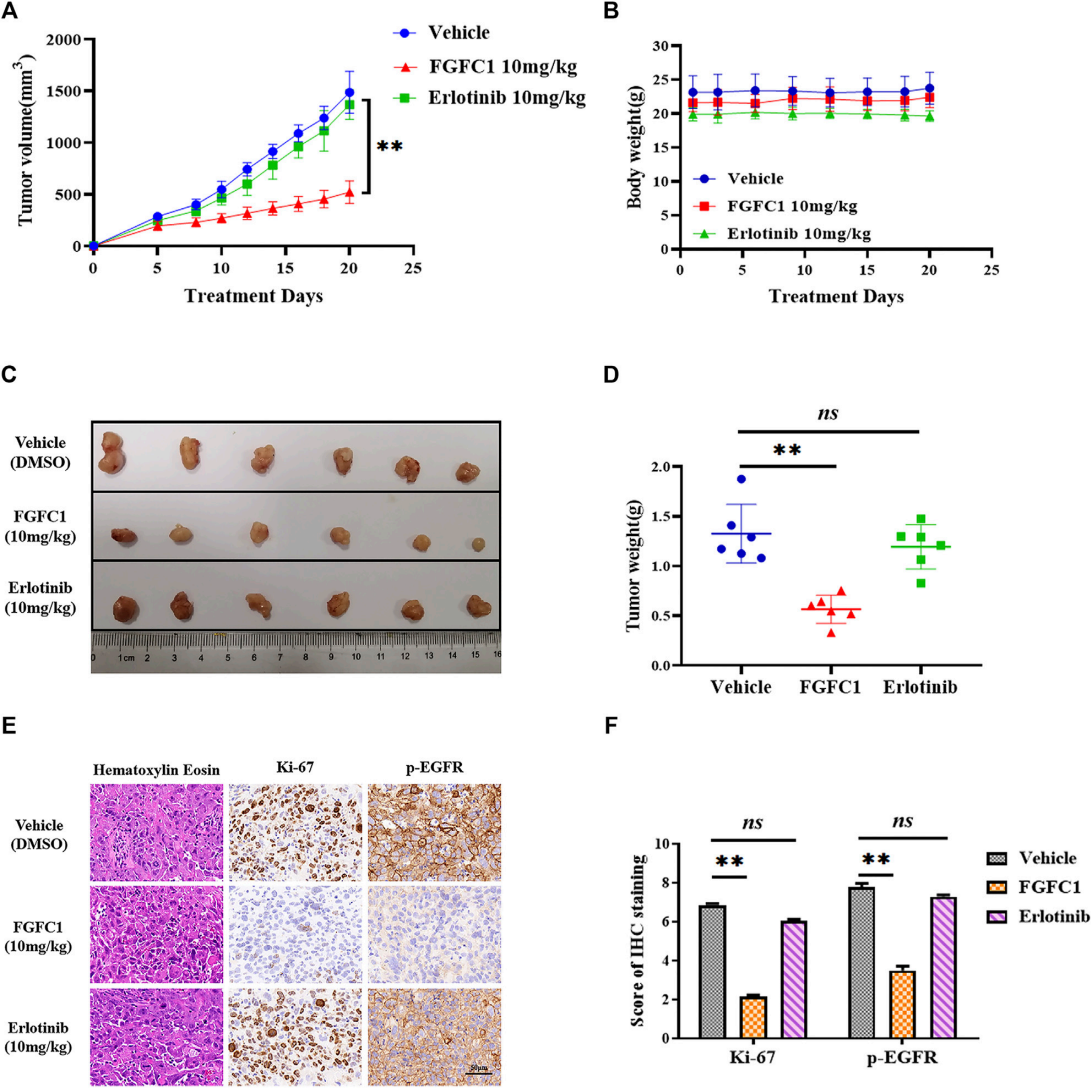
FGFC1 inhibited the growth of H1975 cell-derived tumor *in vivo*. H1975 cells were subcutaneously inoculated into the flank of nude mice. The mice were randomized into three groups. **(A)** Tumor volume was measured by caliper once every 3 days when the tumor reached approximately 60 mm^3^ in size. **(B)** 7 days after H1975 cells implantation, mice were treated with vehicle (5% DMSO in PBS, ip), FGFC1 (10 mg/kg, ip) and erlotinib (10 mg/kg, ip) once a day for 21 consecutive days. The body weight was quantified in each group. **(C)** The representative stripped images of the tumor entity after being treated with vehicle, FGFC1 and erlotinib for 21 days. **(D)** The scatter plot summarized the weight of the tumors. **(E)** FGFC1 decreased the expression of p-EGFR, Ki-67 *in vivo*. The expression of p-EGFR and Ki-67 in tumor tissues from nude mice was assessed by immunohistochemistry (400×) (scale bar = 50 μm). **(F)** The IHC score of p-EGFR and Ki-67 was quantified by the IRS system (*n* = 10 fields of view). All data were represented as the means ± SD for at least three independent experiments. (*p < 0.05 and **p < 0.01 vs the DMSO control).

In order to further determine whether or not FGFC1 was able to exert the same anti-cancer effects *in vivo*, we evaluated such effects of FGFC1 *in vivo* by xenograft mice bearing H1975 tumors. The data showed that FGFC1 significantly suppressed subcutaneous tumor growth in nude mice (Figure 6A), but the body weight of mice did not decrease notably (Figure 6B). The average tumor weight in the FGFC1-treated group was obviously lower than that in the control and erlotinib-treated group (Figures 6C,D). In addition, the immunohistochemical analysis indicated that FGFC1 treatment decreased the expression of Ki-67, a marker for tumor cell proliferation, and significantly reduced EGFR phosphorylation in tumor cells which were consistent with the *in vitro* study (Figures 6E,F). In conclusion, the results demonstrated that FGFC1 inhibited NSCLC tumor growth via regulating EGFR signaling pathways.

## Discussion

*EGFR* mutations result in the activation of EGFR and its downstream signaling pathways, which contribute to the progression of many human cancers, including lung cancer (Hsieh et al., 2013). Overexpression of EGFR exists in more than 45% of NSCLC cases, which has been considered as a predictor of low survival rate and poor outcome of NSCLC patients. In recent years, a series of TKIs targeting EGFR have been developed and approved for the treatment of NSCLC patients harboring *EGFR* mutations, such as erlotinib and gefitinib. Unfortunately, the progression of cancer resulting from acquired resistance to EGFR-TKIs is a critical obstacle in the treatment with NSCLC.

Therefore, identification and development of novel drugs targeting *EGFR* T790M with good curative effects and limited side effects have great clinical significance. In the present study, we provided evidence for the first time that FGFC1 possessed selective anti-cancer activity against *EGFR*-mutant PC9 and H1975 NSCLC cells. It is known that H1975 cells harbor the L858R-activating and T790M resistant mutations of *EGFR* and are erlotinib-resistant. Subsequently, we revealed the inhibitory effect mechanism of FGFC1 on H1975 NSCLC cells both *in vivo* and *in vitro*. Our results showed that FGFC1 was a potential target molecule for overcoming T790M resistance.

Inhibiting the proliferation of tumor cells is a critical aspect while determining the approaches to treat cancer (Wang et al., 2018). In this study, we investigated the effects of FGFC1 on different cancer cell lines, including lung cancer, cervical cancer, colon cancer, melanoma, and normal renal epithelial cells. CCK-8 assay demonstrated that FGFC1 revealed a selective cytotoxic effect on NSCLC cells PC9, it was relatively safe for normal renal epithelial cells 293T and other cancer cells (Figures 1B–D). This data indicated that FGFC1 exerted certain selectivity to NSCLC with *EGFR* mutations. Thus, we further explored the effects of FGFC1 on various NSCLC cells with different *EGFR* status. In addition to PC9, a surprising finding was discovered. FGFC1 also exerted significantly inhibitory effect on erlotinib-resistant H1975 NSCLC cells proliferation, while only slightly inhibitory effect on *EGFR* wild type cells A549 and H1299 (Figures 1E–H).

Since cell apoptosis is an important mechanism in the induction of cell death, we subsequently elucidated cellular apoptosis after FGFC1 treatment. We examined whether FGFC1 could induce cell apoptosis of erlotinib-resistant H1975 NSCLC cells. Meanwhile, using *EGFR* wild-type cell lines (A549 and H1299) as a parallel control. Annexin V and PI staining revealed that compared with control cells, FGFC1 selectively induced apoptosis in H1975 erlotinib-resistant NSCLC cells (Figures 2A,B). Consistent with these results, we clarified that the FGFC1 treatment can significantly promote the expression levels of cleaved-PARP, cleaved-caspase-3, and Bax, whereas induced a decrease in the expression levels of Bcl-2 (Figures 2C,D). Thus, our results indicated that FGFC1 could selectively kill the erlotinib-resistant H1975 NSCLC cells.

The mitochondrion is an important organelle involved in cell death (Green and Kroemer, 2004), and it is also a central processor in the processes of energy metabolism, cell signal transduction, and apoptosis regulation (Li et al., 2021). When apoptosis of cells was induced by external factors, a decrease in MMP causes apoptotic factors to release from mitochondria into the cytosol, which further triggers caspase-dependent or caspase-independent apoptotic pathways. Many studies have displayed that triggering mitochondria-mediated apoptosis becomes an available method for therapy of resistant cancer. In addition, mitochondria dysfunction could cause the generation of intracellular ROS (Kim et al., 2021), which is confirmed to be involved in the drug resistance related to EGFR and its downstream pathway (Weng et al., 2018; Zhang et al., 2019). Excessive ROS produced by external stimuli was not conducive to the survival of cells, thus causing the accumulation of ROS and cell apoptosis (Yuan et al., 2019). Moreover, previous reports have corroborated that ROS played an important role in regulating PI3K/Akt/mTOR pathways, which is highly associated with migration, Invasion, and tumorigenesis. Chen et al. (2019) have reported that Glutathione Peroxidase 1 could induce the drug-resistant NSCLC apoptosis through ROS-mediated PI3K/Akt signaling pathway. Wu et al. (2020) have demonstrated that tormentic acid could induce anti-cancer effects in cisplatin-resistant cervical cancer via ROS-mediated PI3K/Akt pathway. Additionally, it also has been proved that chemical-induced ROS generation was mediated by PI3K/Akt signaling pathway. Ji et al. (2019) have indicated that 2′,4′-Dihydroxy-6′-methoxy-3′,5′-dimethylchalcone-induced ROS generation causing cell apoptosis was via suppression PI3K/Akt pathway. Yen et al. (2012) have clarified that Arsenic-induced cell apoptosis was regulated by PI3K/Akt pathway-mediated ROS. Herein, we showed that FGFC1 induced the dysfunction of mitochondrial, leading to the accumulation of intracellular ROS and GSH depletion in H1975 cells (Figures 3A–E). Moreover, NAC preconditioning could alleviate FGFC1-induced ROS accumulation and block the upregulation of intrinsic pro-apoptotic proteins induced by FGFC1, suggesting that FGFC1 triggered apoptosis of erlotinib-resistant H1975 NSCLC cells via inducing the mitochondrial dysfunction and ROS accumulation (Figures 3F–I). Additionally, given the close relationship between ROS and PI3K/Akt pathway (Zhang and Yang, 2013; Zhang et al., 2017; Zhu et al., 2021), then we researched the effect of FGFC1 on this pathway. The results showed that FGFC1 decreased the phosphorylation of Akt, whereas NAC indeed alleviated the inhibition of Akt phosphorylation induced by FGFC1 (Figure 3H), demonstrating that the PI3K/Akt signaling pathway might be involved in the FGFC1-induced cell apoptosis.

The PI3K/Akt signaling, an important pathway downstream of EGFR, has been demonstrated that aberrant activation of it could promote TKI resistance in NSCLC (Toulany and Rodemann, 2015). Therefore, inhibition of the PI3K/Akt pathway might overcome TKI resistance effectively. Here, we showed that FGFC1 could selectively inhibit the expressions of p-EGFR, p-PI3K, p-Akt, and p-mTOR in erlotinib-resistant H1975 NSCLC cells (Figures 4A,B). The LY294002 and FGFC1 combined treatment group was found to have aggravated the accumulation of ROS and cell apoptosis through enhancing the inhibitory effects of FGFC1 on the EGFR/PI3K/Akt/mTOR pathway (Figures 4E–H). The data clarified that FGFC1 inhibited cell viability and induced apoptosis of erlotinib-resistant NSCLC cells via negative regulation of PI3K/Akt/mTOR signaling pathway. However, the precise molecular mechanisms of ROS accumulation regulated by PI3K/Akt pathway still need to be further studied. To identify the direct target of FGFC1, we subsequently conducted molecular modeling and found that FGFC1 had a great potential to target EGFR^T790M/L858R^ (Figures 4I,J). In addition, tumor metastasis is a major contributor to cancer-related deaths, accounting for 90% of deaths (Pritchard et al., 2011; Zeeshan and Mutahir, 2017). Studies have reported that the PI3K/Akt/mTOR pathway activation was associated with enhanced invasive and migratory in NSCLC (Fu et al., 2015; Pérez-Ramírez et al., 2015). Our data verified that FGFC1 could remarkably inhibit the migratory and invasive abilities of H1975 cells, selectively (Figure 5).

Finally, we assessed the anti-cancer effects of FGFC1 *in vivo*. Erlotinib has been used as the first-line TKIs in NSCLC clinical treatments. As parallel control, we compared the anti-cancer effects of the FGFC1 and erlotinib by reduced tumor weight and size when the tumor-bearing animals were treated with them. To our satisfaction, FGFC1 had no significant toxicity effect on mice body weight and tissue histology. FGFC1 (10 mg/kg) showed more potent anti-cancer properties in suppressing H1975 solid tumors compared with vehicle (5% DMSO in PBS) or erlotinib (10 mg/kg) groups, which was consistent with studies *in vitro* (Figure 6). Additionally, immunohistochemical results of study *in vivo* further confirmed that FGFC1 suppressed erlotinib-resistant NSCLC xenograft tumor growth through decreasing p-EGFR and Ki67 expression.

**FIGURE 7:**
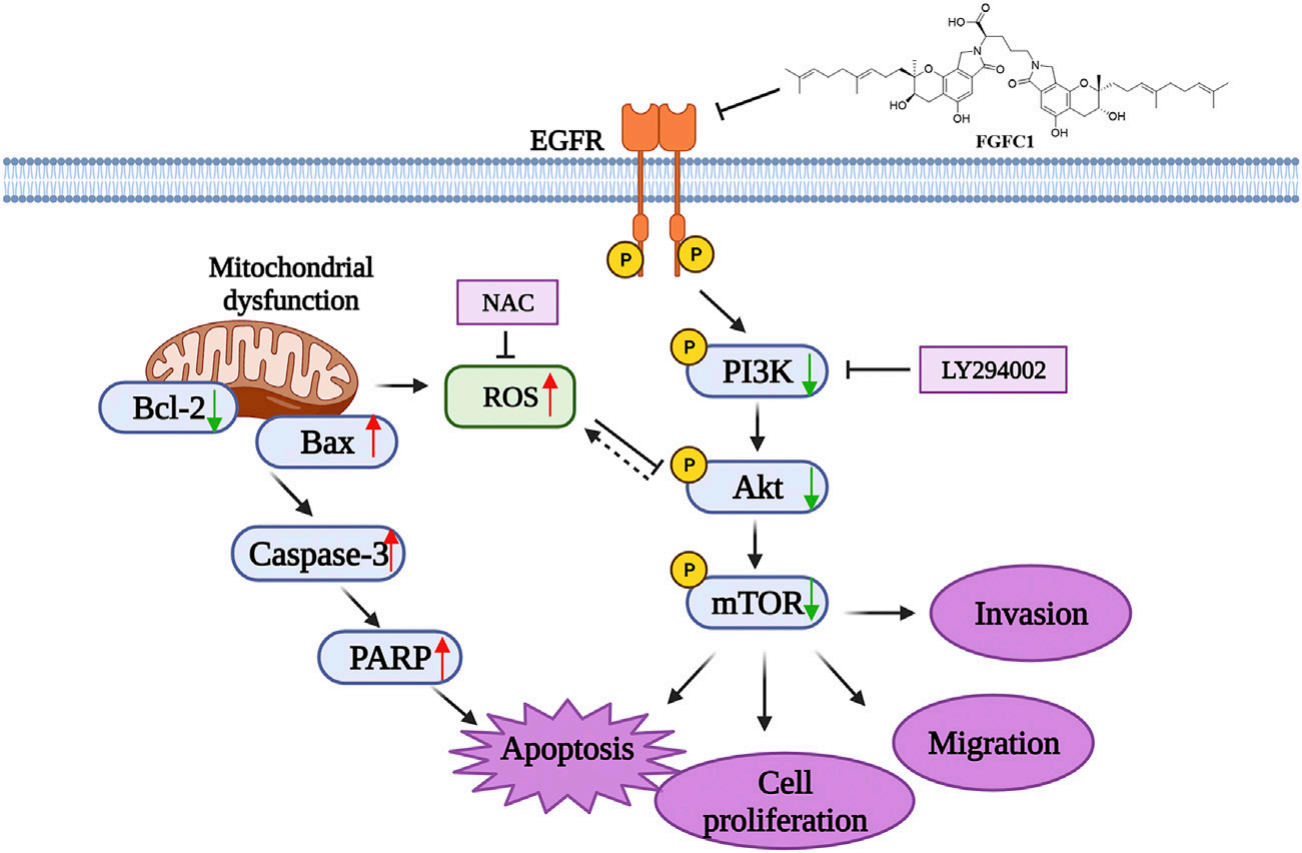
The proposed mechanism of the effect of FGFC1 on NSCLC cells.

Based on our present results, we have proposed an outline of FGFC1 anti-cancer mechanism in Figure 7. Our overall results demonstrated that FGFC1 could effectively interact with mutated *EGFR* and consequently selectively kill erlotinib-resistant H1975 NSCLC cells. Furthermore, FGFC1 can efficiently inhibit the cell viability by leading mitochondrial dysfunction, accumulation of ROS *in vivo*, and down-regulation of EGFR/PI3K/Akt/mTOR signaling pathway. Therefore, FGFC1 might be an effective anti-cancer candidate in the clinical settings of erlotinib-resistant NSCLC. The preliminary molecular mechanism of FGFC1 on the erlotinib-resistant NSCLC was revealed and provided a sound rationale for future investigation in the clinical setting.

## Data Availability

The original contributions presented in the study are included in the article/Supplementary Material, further inquiries can be directed to the corresponding authors.
