# Supplementary material for: FGFC1 Selectively Inhibits Erlotinib-Resistant Non-Small Cell Lung Cancer via Elevation of ROS Mediated by the EGFR/PI3K/Akt/mTOR Pathway
Source: Front Pharmacol. 2022 Jan 19;12:764699. doi: 10.3389/fphar.2021.764699 (PMC8807551; doi:10.3389/fphar.2021.764699)
Supplement: Supplementary file 1 [file DataSheet1.docx]

**
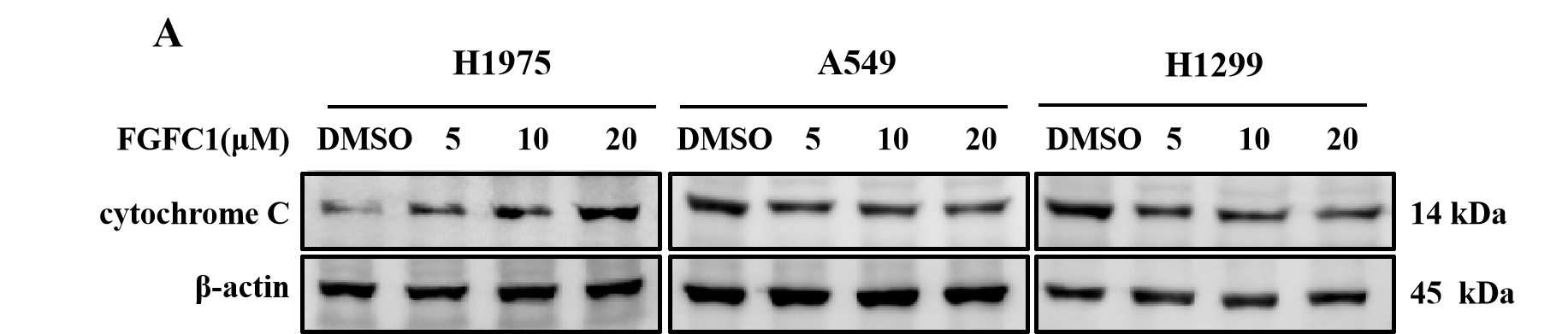
**

**
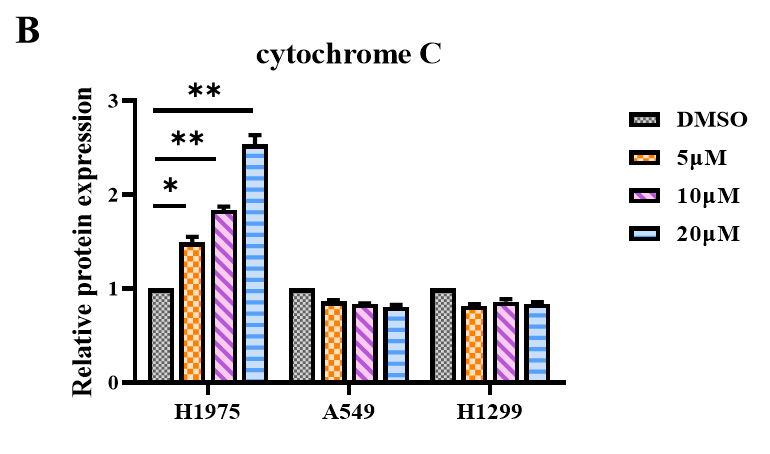
**

**Supplementary Figure 1**, **FGFC1 increased the release of cytochrome C in erlotinib-resistant NSCLC cells.** (A) H1975, A549 and H1299 cells were treated with FGFC1 (0, 5, 10 and 20 µM) for 24 h. The protein level of the cytochrome C was examined by Western blotting. β-actin was detected as the endogenous loading control, accordingly. (B) The statistical result of (A). Data were represented as the mean ± SD for at three independent experiments. **p* < 0.05 and ***p* <0.01 compared with control.
